# Goldenhar syndrome: a cause of secondary immunodeficiency?

**DOI:** 10.1186/1710-1492-8-10

**Published:** 2012-07-02

**Authors:** Serge De Golovine, Shuya Wu, Jill V Hunter, William T Shearer

**Affiliations:** 1 Section of Immunology, Allergy and Rheumatology, Department of Medicine, Baylor College of Medicine, Houston, TX, USA; 2 Texas Children’s Hospital, Houston, TX, USA; 3 Medical Education Department, Driscoll Children’s Hospital, Corpus Christi, TX, USA; 4 Department of Pediatric Radiology, Baylor College of Medicine, Houston, TX, USA; 5 Section of Allergy and Immunology, Department of Pediatrics, Baylor College of Medicine, Houston, TX, USA

**Keywords:** Goldenhar syndrome, Hemifacial microsomia, Oculo-auriculo-vertebral dysplasia, Recurrent infections, Sinusitis, Otitis, Meningitis, Immunodeficiency

## Abstract

Goldenhar syndrome (GS) results from an aberrant development of the 1^st^ and 2^nd^ branchial arches. There is a wide range of clinical manifestations, the most common being microtia, hemifacial microsomia, epibulbar dermoids and vertebral malformations. We present two cases of GS and secondary immunodeficiency due to anatomical defects characteristic of this disorder. Case 1 (3-year-old female) averaged 6 episodes of sinusitis and otitis media per year. Case 2 (7-year-old female) also had recurrent otitis media, an episode of bacterial pneumonia, and 2 episodes of bacterial meningitis. Their immune evaluation included a complete blood count with differential, serum immunoglobulin levels and specific antibody concentrations, lymphocyte phenotyping, and mitogen and antigen responses, the results of which were all within normal ranges. Both children demonstrated major structural abnormalities of the inner and middle ear structures, retention of fluid in mastoid air cells, and chronic sinusitis by computed tomography. These two cases illustrate how a genetically-associated deviation of the middle ear cleft can cause recurrent infections and chronic inflammation of the middle ear and adjacent sinuses, even meninges, leading to a greatly reduced quality of life for the child and parents.

## Background

**Figure 1:**
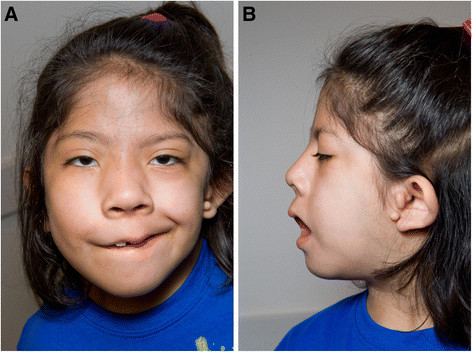
** 7-year-old girl with Goldenhar Syndrome (Case Presentation 2): A, frontal view; B, left lateral view.** The left lower side of the face is underdeveloped and the external helix of the ear is misshapen with external ear tags.

Goldenhar syndrome (GS), also known as hemifacial microsomia, oculo-auriculo-vertebral anomaly, dysplasia or spectrum, results from an aberrant development of the 1^st^ and 2^nd^ branchial arches [1]. There is a wide range of clinical manifestations, the most common being microtia, hemifacial microsomia, pre-auricular skin tags, epibulbar dermoids, and vertebral malformations (Figure 1). Other skeletal abnormalities and ocular, cardiac and renal anomalies have been described [2-4]. This condition has a prevalence ranging from 1:3500 to 1:7000 live births with a male to female ratio of 3:2 [2,5]. To our knowledge, there have been no case reports of GS in association with immune deficiencies.

The etiology of GS remains unknown. It has been suggested that a vascular insult and/or neural crest pathology during a critical time of embryogenesis could account for this syndrome [6]. Some cases have been reported to have autosomal dominant, autosomal recessive and multifactorial inheritance patterns, however, most GS cases are sporadic [7-9]. No chromosomal errors or gene mutations have yet been detected in GS.

These two case presentations emphasize the importance of anatomical deviations of middle ear and sinus structures that impair proper drainage of secretions as seen in GS that lead to a condition of secondary immunodeficiency.

## Case presentation 1

A 3-year-old female child with GS was referred to the Allergy and Immunology Clinic at Texas Children’s Hospital for evaluation of recurrent infections. According to the mother, the patient’s infections were diagnosed at different private clinics by either pediatric or ear, nose, and throat physicians with an average of 6 bouts of sinusitis and otitis media per year all requiring antibiotics. The presence of otitis media and sinusitis was diagnosed on the basis of fever, ear pain, and fetid greenish discharge from the nostrils of the child. During the disease free intervals, the patient had clear nasal discharge.

The diagnosis of GS was based on left microtia, bilateral external ear canal stenosis, hypoacusia, pre-auricular skin tags, left hemifacial microsomia, multiple ventricular septal defects, and hypersegmented C2 and C3 vertebrae. Her past surgical history included, bilateral pre-auricular tag removal in May of 2009, right macrostomia repair with musculocutaneous flap transfer in June of 2009, and right myringotomy with tube placement in July of 2010. There was no family history of GS or immune deficiencies.

**Figure 2:**
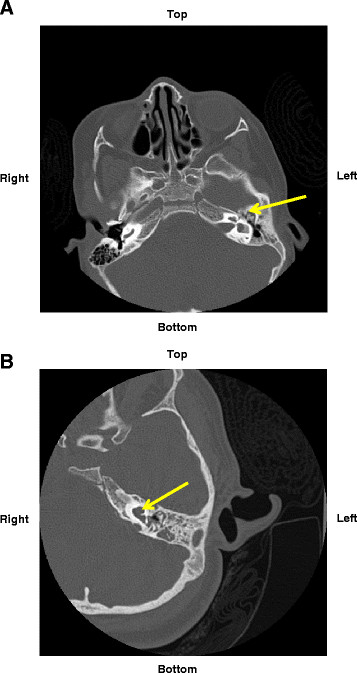
** CT scans of inner ear cavities and adjacent sinuses performed at Texas Children’s Hospital. A**, Case Presentation 1 showing diminished left middle ear cavity and mastoid sinus (yellow arrow); **B**. Case Presentation 2 showing a dysplastic cochlea with an abnormally large basal turn and only partially formed middle and to a lesser extent apical turns (yellow arrow). The vestibule was also dysplastic (not shown).

The physical examination revealed a 3-year-old girl with the classic GS findings. She was in the 5^th^ and 10^th^ percentiles for weight and height, respectively. She had bilateral malformed ears with left ear canal stenosis; a right tympanostomy tube in the right tympanic membrane with white discharge; and a pronounced downward slant of the right side of the mouth. A computed tomography (CT) scan of the sinuses showed the left middle ear cavity and mastoid being diminished in size and partially opacified. (Figure 2A)

Immune evaluation included a complete blood count (CBC) with differential cell count, serum immunoglobulin levels and specific antibody concentrations (measured at 33 months of age, 15 months after vaccination with diphtheria, tetanus, and acellular pertussis vaccine, and pneumococcal 13-valent vaccine), lymphocyte phenotyping, and mitogen and antigen responses, the results of which were all within normal ranges (Tables 1, 2).

**Table 1:** Immune Cells, Serum Immunoglobulin, and Specific Antibodies to Antigens in 2 patients with Goldenhar Syndrome

	**Patient 1**	**Patient 2**
	**3 y**	**7 y**
**WBC** cells/μL	8930 (5,200–12,000)	14,080 (5,000–14,500)
**Polymorphonuclear leukocytes cells/μL**	5340 (1,200–5,200)	8054 (1,500–8,563)
**Lymphocytes** cells/μL	2480 (2300–5400)	4731 (2,112–8,589)
**IgG** (mg/dL)	1140 (546–1454)	1174 (635–1284)
**IgM** (mg/dL)	102 (27–176)	209 (44–190)
**IgA** (mg/dL)	60.7 (59–269)	135 (32–191)
**Diphtheria Toxoid IgG** (IU/mL)	1.5 (≥0.10)	8.8 (≥0.10)
**Tetanus Toxoid IgG** (IU/mL)	1 (≥0.10)	0.2 (≥0.10)
**Streptococcus Pneumoniae Type 1** (μg/mL)	4.11 (≥1.3)	8.27 (≥1.3)
**Streptococcus Pneumoniae Type 3** (μg/mL	3.35 (≥1.3)	0.51 (≥1.3)
**Streptococcus Pneumoniae Type 4** (μg/mL)	8.91 (≥1.3)	9.31 (≥1.3)
**Streptococcus Pneumoniae Type 5** (μg/mL)	22.52 (≥1.3)	4.15 (≥1.3)
**Streptococcus Pneumoniae Type 6B** (μg/mL)	14.03 (≥1.3)	≥ 37.83 (≥1.3)
**Streptococcus Pneumoniae Type 7F** (μg/mL)	0.7 (≥1.3)	3.75 (≥1.3)
**Streptococcus Pneumoniae Type 8** (μg/mL)*	0.62 (≥1.3)	0.64 (≥1.3)
**Streptococcus Pneumoniae Type 9N** (μg/mL) *	2.16 (≥1.3)	1.65 (≥1.3)
**Streptococcus Pneumoniae Type 9V** (μg/mL)	3.77 (≥1.3)	4.25 (≥1.3)
**Streptococcus Pneumoniae Type 12F** (μg/mL) *	0.56 (≥1.3)	0.43 (≥1.3)
**Streptococcus Pneumoniae Type 14** (μg/mL)	12.14 (≥1.3)	14.51 (≥1.3)
**Streptococcus Pneumoniae Type 18C** (μg/mL)	9.85 (≥1.3)	14.35 (≥1.3)
**Streptococcus Pneumoniae Type 19F** (μg/mL)	>59.27 (≥1.3)	29.2 (≥1.3)
**Streptococcus Pneumoniae Type 23F** (μg/mL)	7.9 (≥1.3)	7.53 (≥1.3)

Age-related normal ranges in parenthesis are taken from Texas Children’s Hospital, Houston, Texas.

*Antigens not contained in the 13-valent pneumococcal vaccine. Controls of the day were run but not shown here.

**Table 2:** Peripheral Blood Mononuclear Cell Phenotyping of 2 patients with Goldenhar Syndrome

**MONONUCLEAR CELL PHENOTYPES**	**% OF TOTAL GATED POPULATION**		**CELL COUNTS**	
	**PATIENT 1**	**PATIENT 2**	**PATIENT 1**	**PATIENT 2**
	**3 y**	**7 y**	**(#/mm^3) 3 y**	**(#/mm^3) 7 y**
CD45^+^ (Purity)	100.0 (100)	100.0 (100)	2483 (N/A)	1726 (N/A)
CD3^-^CD56,16^+^	12.5 (3.0–18.6)	17 (5–21)	311 (123–785)	815 (128–474)
CD56+	10.1 (6.2–23.2)	17 (6–23)	251 (137–478)	815 (137–478)
CD2^+^	64.6 (69.8–93.2)	64 (70–93)	1604 (884–2800)	3068 (884–2800)
CD3^+^ (Total)	57.5 (58.0–74.0)	58 (56–76)	1427 (1656–3841)	2781 (991–2997)
CD3^+^CD4^+^	39.6 (28.0–47.2)	33.9 (25–48)	983 (871–2379)	1625 (635–1620)
CD3^+^CD8^+^	15.5 (16.0–31.8)	19 (16–43)	384 (518–1433)	911 (293–1221)
CD19^+^	28.7 (12.8–30.6)	24 (11–28)	713 (421–1397)	1151 (249–865)
CD20^+^	27.7 (2.7–24.9)	23 (3–25)	688 (59–457)	1103 (59–457)
CD19^+^CD27^+^	2.1 (1.0–5.2)	1.9 (1–5)	52 (19–131)	91 (19–131)
HLA-DR^+^	36.3 (8.9–36.4)	29 (9–36)	901 (177–692)	1390 (59–457)
CD4^+^CD45RA^+^	27.6 (3.3–32.9)	24 (3–33)	686 (134–969)	1151 (134–969)
CD4^+^CD45RO^+^	10.8 (15.6–41.8)	10 (16–42)	269 (301–919)	479 (301–919)
CD4/CD8 Ratio	2.56 (0.72–2.94)	1.78 (0.69–2.63)	N/A	N/A

Age-related normal ranges in parenthesis are taken from Allergy and Immunology Laboratory, Texas Children’s Hospital, Houston, Texas. Controls of the day were run but not shown here.

## Case presentation 2

A 7-year-old female child who was known to have GS and bilateral sensorineural hearing loss was referred to the Allergy and Immunology Clinic at Texas Children’s Hospital for evaluation of recurrent infections. She had an episode of bacterial meningitis 2 months before presentation. At that time she was initially taken to an outside hospital due to a 1 day history of vomiting, fever of 38.9° C, and lethargy. With the suspicion of bacterial meningitis, she was empirically treated with vancomycin, ceftriaxone, and decadron 3 hours before a lumbar puncture revealed purulent cerebral spinal fluid (CSF). CSF analysis was consistent with bacterial meningitis, showing a cloudy fluid with 2,815 white blood cells/mL (WBC) including 84% segmented cells 9% lymphocytes, 7% monocytes, 15 red blood cells/μL (RBC), a glucose of less than 5 mg/dL, and a protein of 505 mg/dL. Analysis of the CSF proved negative for pneumococcal and meningococcal antigens, the CSF gram stain did not reveal organisms, and the culture remained negative. A CT scan of the head was also performed and was reported as normal.

She was subsequently transferred to Driscoll Children’s Hospital, Corpus Christi, Texas for further management of her meningitis where a CBC showed a WBC count of 37,900 cells/μL, with 14% banded cells, 79% segmented cells, 2% lymphocytes, and 1% atypical forms. The hemoglobin concentration was 9.3 gm/dL, and the platelet count was 452,000 cells/μL. On hospital day 2, the patient developed left-sided neck pain and maintained her head tilted towards the left side, raising concern for spinal/epidural abscess. A CT scan of her neck failed to show a spinal/epidural abscess, but was positive for a C2-C3 cervical fusion and a small arachnoid cyst at the left posterior cranial fossa. In addition, partial opacification of the left mastoid air cells and left middle ear cavity as well as some fluid at right mastoid air cells was found (not shown). Of note, the patient had a right cochlear implant since age 3 years. A careful review of her CT scan at the time of her meningitis showed no evidence of inflammatory changes surrounding the right cochlear implant (not shown), making the implant the cause of the meningitis much less likely. The patient gradually improved, and was discharged home after receiving 10 days treatment of ceftriaxone.

The patient’s diagnosis of GS was based on her characteristic facial features (Figure 1). Genetic tests were performed but did not reveal any abnormalities. Her past medical history was significant for recurrent ear infection diagnosed by pediatricians, around 4–5 episodes per year and persistent ear fluid bilaterally, which required tympanostomy tube placements and tonsillectomy and adenoidectomy at age of 5. After the surgeries, she continued to have ear infections but with less frequency. She had one previous episode of meningitis secondary to *Streptococcus pneumoniae* at 11 months of age, for which she was hospitalized for 10 days and received IV antibiotics. She was again hospitalized for pneumonia requiring IV antibiotics at 2 years of age.

At the time of her visit to the Allergy and Immunology Clinic at Texas Children’s Hospital, she was in the 75^th^ and 50^th^ percentiles of weight and height, respectively. She had left hemifacial microsomia and bilateral malformed ears with multiple ear tags. A CT scan of the temporal bone performed 2 months prior to her meningitis was reviewed and revealed inner ear malformations including bilateral labyrinthine dysplasia and significant hypoplasia of the right internal auditory canal. The left cochlea was noted to be dysplastic (Figure 2B).

Her routine childhood immunizations were up to date(diphtheria, tetanus, and acellular pertussis, and the pneumococcal 13-valent vaccine given between 4 and 6 years), and she received the meningococcal vaccine and another pneumococcal 13-valent vaccine prior to discharge from Driscoll Children’s Hospital. Her immune evaluation included a CBC with differential count, immunoglobulin levels, specific antibody titers (measured at 7 years of age, 2 months after vaccination with the 13-valent pneumococcal vaccine), lymphocyte phenotyping, mitogen and antigen responses, all reported to be within normal ranges (Tables 1 and 2).

The majority of secondary immunodeficiencies, such as those illustrated by these two case presentations, occur as the result of diseases or conditions extrinsic to the immune system such as malnutrition, immunosuppressive agents, surgery and trauma, extremes of age, environmental factors, and genetic syndromes. Correction of the primary defect usually leads to prevention or reversal of the related immune defect [10].

In addition, the combination of anatomical defects and the capability of many pathogens of interfering with a variety of immunological defenses create a vicious cycle of epithelium-immune dysfunction with decrease in pathogen clearance [10].

Examples of these anatomical defects are seen in patients with Eustachian tube dysfunction and congenital anomalies such as Down syndrome, cleft palate, Townes and oral-facial-digital syndromes, and the Di George anomaly that are known to have a higher incidence of recurrent or persistent otitis media. [11-14]. The basis for these recurrent infections in these patients with abnormal otonasopharyngeal anatomy lies in their inability to coordinate proper drainage of secretions and bacteria particularly when tissues are acutely or chronically inflamed. With Down syndrome, stenotic ear canals contribute to a marked increase in middle ear infusions [12]. In the case of the partial Di George anomaly, where patients have mild forms of T cell deficiency, bacterial and viral infections are prolonged. Abnormal formations of the palate predispose the Di George patient to recurrent upper airway infections. [14]. Children with cochlear dysplasia may develop a CSF fistula leading to recurrent meningitis [15].

## Conclusions

In summary, these two case presentations illustrate how a genetic deviation of the middle ear cleft can cause recurrent infections of the middle ear and adjacent sinuses, even meninges, leading to a greatly reduced quality of life for the child and parents. The lack of proper drainage of the middle ear and sinuses are most likely the cause of repetitive infections despite normal immune responses. Surgery and trauma that produce anatomical dislocations are well accepted causes of secondary immunodeficiency leading into acute and chronic infection due to the interruption of normal flow of body secretions. Similar in principle, we believe, is the lack of upper respiratory secretion clearance in these children with Goldenhar syndrome.

Accordingly, we propose these two cases to be examples of secondary immunodeficiency related to anatomical defects of the middle ear and auditory canals, where an immune evaluation excluded primary immune deficiencies. As in all of the secondary immune deficiencies, we recommend correction of the causative illness, which in this case would include surgical correction of the anatomical defect with improvement of sinus drainage and simultaneous antibiotic eradication of the pathogens.

## Consent

Consent to publish photographs, scan images, and medical information were given by the parents on behalf of their children.

## Abbreviations

GS, Goldenhar Syndrome.

## Competing interests

The authors declare that they have no competing interests.

## Authors’contributions

SDG and SW gathered clinical information and wrote the manuscript; JVH reviewed the CT scans of the middle ear left and adjacent sinuses; and WTS supervised the activities of SDG and SW and assisted with writing and revising the manuscript. All authors read and approved the final manuscript.

## Authors’ information

Dr. Serge DeGolovine is senior resident in the allergy and immunology training program at Baylor College of Medicine in Houston, TX, USA; Dr. Shuya Wu is senior resident in the pediatric training program at Driscoll Children’s Hospital in Corpus Christi, TX, USA; Dr. Jill V. Hunter is professor of pediatric radiology at Baylor College of Medicine, Houston, TX, USA; and Dr. William T. Shearer is professor of pediatrics and immunology and director of the allergy and immunology training program at Baylor College of Medicine, Houston, TX, USA.
